# Investigation of an international outbreak of multidrug-resistant monophasic *Salmonella* Typhimurium associated with chocolate products, EU/EEA and United Kingdom, February to April 2022

**DOI:** 10.2807/1560-7917.ES.2022.27.15.2200314

**Published:** 2022-04-14

**Authors:** Lesley Larkin, Maria Pardos de la Gandara, Ann Hoban, Caisey Pulford, Nathalie Jourdan-Da Silva, Henriette de Valk, Lynda Browning, Gerhard Falkenhorst, Sandra Simon, Raskit Lachmann, Rikard Dryselius, Nadja Karamehmedovic, Stefan Börjesson, Dieter van Cauteren, Valeska Laisnez, Wesley Mattheus, Roan Pijnacker, Maaike van den Beld, Joël Mossong, Catherine Ragimbeau, Anne Vergison, Lin Thorstensen Brandal, Heidi Lange, Patricia Garvey, Charlotte Salgaard Nielsen, Silvia Herrera León, Carmen Varela, Marie Chattaway, François-Xavier Weill, Derek Brown, Paul McKeown

**Affiliations:** 1 Gastrointestinal Infections and Food Safety (One Health Unit), UK Health Security Agency, London, United Kingdom; 2 Institut Pasteur, Université Paris Cité, Centre National de Référence des E. coli, Shigella et Salmonella, Unité des Bactéries pathogènes entériques, Paris, France; 3 Sante Publique France, Direction des Maladies Infectieuses Unité EAZ, Paris, France; 4 Clinical and Protecting Health Directorate, Public Health Scotland, Glasgow, United Kingdom; 5 Robert Koch Institute, Department of Infectious Disease Epidemiology FG 35 - Gastrointestinal Infections, Zoonoses and Tropical Infections, Berlin, Germany; 6 Robert Koch Institute, Department of Infectious Diseases, Unit for Enteropathogenic Bacteria and Legionella / National Reference Centre for *Salmonella* and other Bacterial Enterics, Wernigerode, Germany; 7 Public Health Agency of Sweden, Unit for Zoonoses and Antibiotic Resistance, Stockholm, Sweden; 8 Public Health Agency of Sweden, Unit for laboratory surveillance of bacterial pathogens, Stockholm, Sweden; 9 Epidemiology of infectious diseases, Department of Epidemiology and Public Health, Sciensano, Brussels, Belgium; 10 National Reference Centre for *Salmonella* and *Shigella*, Sciensano, Brussels, Belgium; 11 National Institute for Public Health and the Environment (RIVM), Centre for Infectious Disease Control, Bilthoven, Netherlands; 12 Health Inspection, Health Directorate, Luxembourg; 13 Laboratoire National de Santé, Epidemiology and Microbial Genomics, Dudelange, Luxembourg; 14 Department of Infection Control and Preparedness, Norwegian Institute of Public Health, Oslo, Norway; 15 HSE -Health Protection Surveillance Centre, Dublin, Ireland; 16 ECDC Fellowship Programme, Field Epidemiology path (EPIET), European Centre for Disease Prevention and Control (ECDC), Stockholm, Sweden; 17 Instituto de Salud Carlos III. Centro Nacional de Microbiología, Madrid, Spain; 18 Instituto de Salud Carlos III. CIBER epidemiología y salud pública. Madrid, Spain; 19 Specialist Scientific Reference Service (Salmonella), Gastrointestinal Bacteria Reference Unit, UK Health Security Agency, London, United Kingdom; 20 Scottish Microbiology Reference Laboratories, Glasgow, United Kingdom

**Keywords:** Monophasic *Salmonella* Typhimurium, outbreak, multi-country collaboration, chocolate products, descriptive epidemiological evidence, whole genome sequencing, core-genome multi locus sequence typing, antimicrobial resistance profile

## Abstract

An extensive multi-country outbreak of multidrug-resistant monophasic *Salmonella* Typhimurium infection in 10 countries with 150 reported cases, predominantly affecting young children, has been linked to chocolate products produced by a large multinational company. Extensive withdrawals and recalls of multiple product lines have been undertaken. With Easter approaching, widespread product distribution and the vulnerability of the affected population, early and effective real-time sharing of microbiological and epidemiological information has been of critical importance in effectively managing this serious food-borne incident.

In February 2022, a small five-single nucleotide polymorphism (SNP) single linkage cluster of eight cases of infection with monophasic *Salmonella enterica* subsp. *enterica* serotype Typhimurium (1,4,5,12:i:-) eBG 1, sequence type (ST) 34 was identified in the United Kingdom (UK). The cluster was unusual, with all but one reported case younger than 10 years, and the strain demonstrated genotypic markers of an unusual antimicrobial resistance pattern not commonly seen in livestock, food or human disease cases in the UK. The cluster was not closely related to any other UK strains of monophasic *S.* Typhimurium.

Exploratory interviews using an open-ended, anthropological approach (not binary yes/no questions) were undertaken with the parents/guardians of five cases in England for hypothesis generation. Subsequently, a targeted questionnaire to refine hypotheses identified through the exploratory interviews was used, confirming a strong signal for a specific brand of chocolate products.

## Coordinated multi-country epidemiological investigations

Following the UK’s notification on the European Centre for Disease Prevention and Control (ECDC) EpiPulse Food and Waterborne Diseases (FWD) platform on 17 February 2022, and an Early Warning and Response System (EWRS) alert on 25 March, Germany, Sweden, France, the Netherlands and subsequently Luxembourg, Norway, Ireland, Belgium and Spain reported confirmed or probable cases in their respective countries. 

### Case definition

The agreed European Union (EU) case definition for confirmed cases was laboratory-confirmed monophasic *S.* Typhimurium with symptom onset on or after 1 October 2021 and belonging to the same five SNP single linkage cluster by SNP typing or cases who clustered within five allelic differences of another confirmed outbreak strain by core genome multilocus sequence typing (cgMLST) analysis or shared the same HC5_296366 by the EnteroBase HierCC scheme [1]. This definition therefore depended on the whole genome sequencing (WGS) methodology used at the national level in each country (i.e SNP typing or cgMLST analysis). Probable cases were those with laboratory confirmation of monophasic *S.* Typhimurium with symptom onset on or after 1 October 2021 and phenotypic antimicrobial resistance (AMR) results consistent with the outbreak strain or a multilocus variable number tandem repeat analysis (MLVA) profile 3–11–14-NA-0211. 

### Detected cases in the EU/EEA and United Kingdom

**Figure 1:**
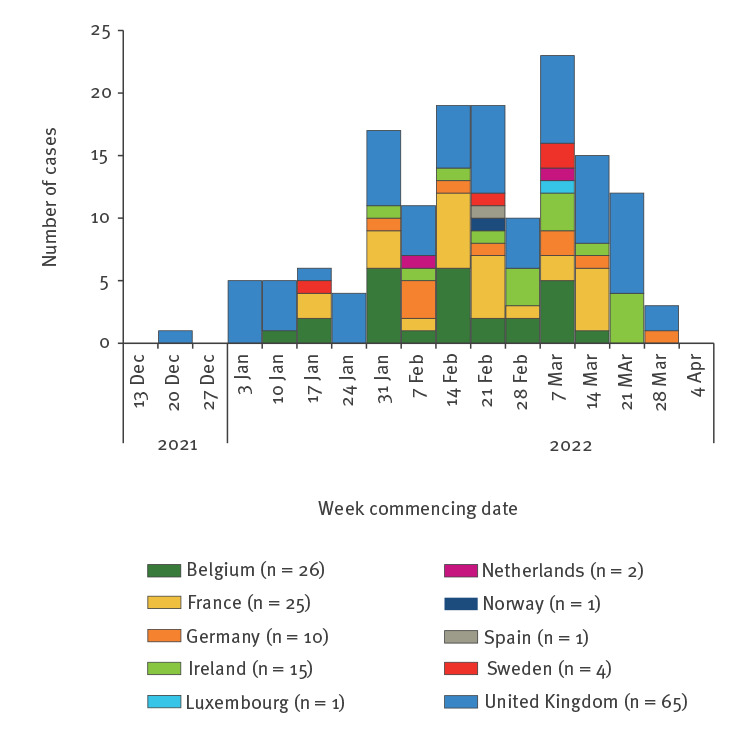
Distribution of confirmed and probable salmonellosis outbreak cases by week and country and by date of onset^a,b^, EU/EEA and UK, up to 10 April 2022 (n = 150)

EEA: European Economic Area; EU: European Union; UK: United Kingdom.

^a^ Case onset date (n=108) is not consistently available for all reported cases, therefore date of sampling has been used where case onset date is unavailable (n=39) or date of receipt at reference laboratory where both onset and sampling dates are unknown (n=3).

^b^ Probable cases were reported by Belgium (n=19) and Germany (n=2) (data as of 13 April).

**Figure 2:**
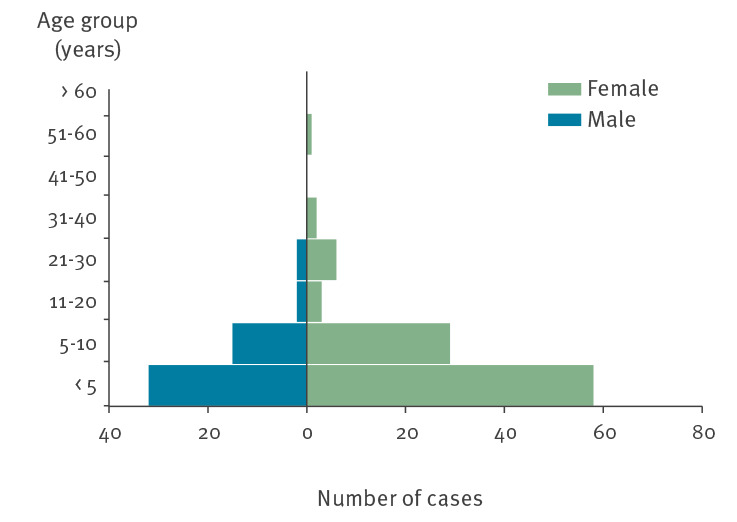
Distribution of confirmed and probable salmonellosis outbreak cases, by age group and sex, EU/EEA and UK, up to 10 April 2022 (n = 150)

EEA: European Economic Area; EU: European Union; UK: United Kingdom.

By 10 April 2022, a total of 150 confirmed and probable cases were identified across nine EU/European Economic Area (EEA) countries and the UK, with case sampling dates ranging from 21 December 2021 (the first UK case) to 28 March 2022 (Figure 1). Descriptive epidemiological investigations demonstrated cases ranged in age from 8 months to 56 years, but were predominately under the age of 10 years (n = 134; 89%) and disproportionately female (n = 99; 66%) (Figure 2). The hospitalisation rate was 42% of cases for whom information was available (116 cases with 49 hospitalised) – higher than that usually reported in salmonellosis outbreaks [2] and for individual cases of infection with *S*. Typhimurium [3]. While this is probably also influenced by the demographic characteristics of those affected, this is a possible indicator of increased clinical severity of infection in this outbreak. 

Multi-country collaboration through teleconferences and sharing of information between public health agencies and reference laboratories indicated that cases in affected countries commonly reported consumption of a specific brand of chocolate products. Overall, of 101 case interviews carried out across the 10 affected countries, 88 cases (87%) confirmed consumption of these products. The most commonly consumed product was Product A, marketed primarily for children in the age group 3–10 years, but multiple other product types were also reported (Table ).

**Table:** Summary of exposure to chocolate produced by the implicated company derived from interviews with confirmed and probable salmonellosis outbreak cases, EU/EEA and UK, up to 10 April 2022 (n = 101)

Country	Number of case interviews	Brand product A	Brand product B	Brand product C	Brand product D	Brand products E–J	Brand products unspecified	Other company products
Belgium	22	19	13	8	8	2	0	6
France	21	12	5	8	0	3	2	9
Germany	7	4	3	2	2	7	0	1
Ireland	11	1	1	0	1	0	6	0
Luxembourg	1	1	0	0	0	0	0	0
Netherlands	2	2	0	0	0	0	0	0
Norway	1	1	0	0	0	0	0	0
Spain	1	0	0	1	0	0	0	0
Sweden	4	2	0	0	0	0	2	0
UK	31	22	12	0	2	5	0	1
**Total**	**101**	**64**	**34**	**19**	**13**	**17**	**10**	**17**

EEA: European Economic Area; EU: European Union; UK: United Kingdom.

## Microbiological investigations

**Figure 3:**
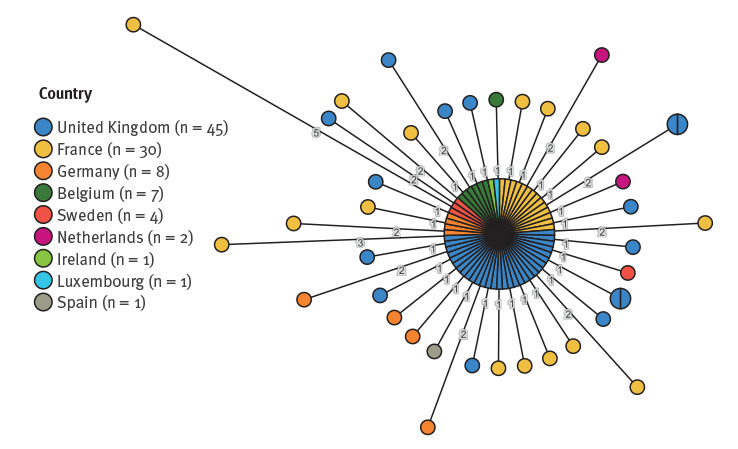
Genomic clustering of the monophasic *S.* Typhimurium isolates (HC5_296366) available on EnteroBase, EU/EEA and UK, up to 13 April 2022 (n = 99)

Source: EnteroBase (https://enterobase.warwick.ac.uk).

The isolates from confirmed cases reported in EU/EEA countries and the UK demonstrated limited genetic diversity. A minimal spanning tree was produced using the cgMLST V2 + HierCC V1_Tree algorithm [4] (Figure 3), including human isolates from nine countries, one per case (n = 99), that were available on EnteroBase (https://enterobase.warwick.ac.uk) and belonged to HC5_296366. The majority of isolates (n = 59; 60%) were genetically indistinguishable (0 allelic differences), with a maximum allelic difference across the cluster of 8 and all isolates having ≤ 5 allelic differences when compared with at least one other isolate in the cluster. Analysis of the UK outbreak cluster alone indicated very limited genetic diversity across the cluster with the average distance between isolates being less than one SNP. 

cgMLST minimal spanning tree of all *Salmonella* isolates of human origin available on EnteroBase in association with HC5_296366 according to country of isolation (generated on 13 April 2022).

Previous analyses on isolates posted on EnteroBase had identified several non-human isolates that fell into the same cgMLST HC5_296366 cluster and had 0 allelic differences compared with the cgMLST profile of the predominant human outbreak case isolates (data not shown). Subsequent enquiries revealed that these isolates were obtained through food business operator sampling of the processing equipment (the buttermilk ingredient tanks) carried out on 15 December 2021 and again in January 2022 in one of the implicated company’s manufacturing facilities. At least 10 implicated product samples, derived from cases’ homes, have been sampled in four countries, with negative results to date.

Notably, the *Salmonella* outbreak strain has an uncommon and extensive AMR profile compared with other monophasic *S*. Typhimurium strains circulating in Europe. Analysis of genotypic markers of antimicrobial resistance (AMR) in seven EU/EEA countries and the UK indicated markers against several classes of antimicrobial drugs. Of specific note were genotypic indicators of the presence of the *lnu(F)* gene conferring resistance to lincosamides. Further phenotypic analysis at the National Reference Laboratories in France, Germany, Belgium and the UK according to EUCAST guidelines [5,6] confirmed resistance to: penicillins (*bla*_TEM-1_), aminoglycosides (streptomycin, spectinomycin, kanamycin, gentamicin *strA-strB*, *ant(3”)_Ia* and *aac(3)_IId*), phenicols (*cmlA1*, *floR*), sulfonamides (*sul3*, perhaps *sul2*), trimethoprim (*dfrA12*) and tetracyclines (*tetA/B and tetM*). There was some variability of the outbreak strain's AMR profile, possibly related to the presence of multiple or chimeric plasmids, warranting further investigation to confirm.

## Outbreak control measures

Food chain investigations in the affected countries indicated that the majority of the products implicated in the epidemiological investigations were produced predominantly at a single production site in Belgium. This was the same facility from which the outbreak strain had been identified in December 2021 in the processing equipment.

Based on the strong descriptive epidemiological evidence implicating these products in this outbreak, on the identified food chain links and on evidence of contamination previously identified at production, risk management actions were taken in all affected countries, including withdrawal of all product lines produced in the implicated production facility and extensive product recalls, supported by news alerts and advice for consumers, starting with the first recall on 2 April in the UK and Ireland and extended to other countries shortly after. Extended recalls were carried out from 7 April as the investigations in a number of countries progressed, resulting in further evidence to support these recalls. On 8 April 2022, Belgian authorities stopped production at the facility in Belgium and, following the European Rapid Alert System Food and Feed (RASFF) alert notifications (RASFF 2022.1799), the World Health Organization/Food and Agriculture Organization International Food Safety Authorities Network (INFOSAN) also issued a global alert on 10 April notifying 77 countries and territories to which distribution of the implicated products had been established to initiate a global recall (see the Supplement for a non-exhaustive list of country-specific recall notices).

As at 10 April 2022, investigations are still ongoing to define specific national supply chains for the implicated products and common sourcing of raw ingredients. Root cause analysis for the outbreak is also ongoing to determine whether the outbreak was caused by a contaminated ingredient or another (potentially multi-strain) source of contamination.

## Discussion

Historically, chocolate-associated outbreaks have been protracted and usually of large scale, probably reflecting both the long shelf life of chocolate and the long survival of *Salmonella* in these products, as well as difficulties in detecting and resolving such outbreaks [7-9]. Previous investigations have resulted in recovery of only small amounts of *Salmonella* bacteria from sampling of chocolate products, suggesting that contamination in chocolate may be difficult to detect in product sampling, as well as difficult to mitigate through routine food hygiene procedures [7]. It has been suggested that the high fat content of chocolate may have a protective effect for the bacteria [10,11], including against gastric acid, and possibly altering the functional infective dose of *Salmonella,* resulting in clinically severe disease from exposure to only very low levels of contamination [12,13].

By 10 April, this outbreak involved at least 150 reported cases in nine EU/EEA countries and the UK. Owing to known under-reporting of *Salmonella* surveillance systems and the varying sensitivities of microbiological techniques used across countries, the scale of the outbreak is certainly underestimated, especially considering that very high volumes of the implicated chocolate products are consumed in the EU/EEA and the UK. However, while the period between initial detection of the outbreak in the UK and subsequent control measures taken at the international level spanned a duration of more than 2 months, once definitive epidemiological links with the implicated product were made, control actions followed rapidly. Indeed, compared with previous outbreaks of salmonellosis associated with chocolate products, the duration of this investigation was relatively short [7,14-16].

The field of infectious disease epidemiology for *Salmonella* has been considerably impacted by the adoption of next generation sequencing technologies combined with novel epidemiological approaches such as iterative open-ended interviewing [17]. The increasing use of WGS enables us to detect and resolve outbreaks more quickly, especially where common serovars such as *S*. Typhimurium are involved, allowing consolidation of evidence implicating specific food vehicles of infection at the international level [18-20].

Another notable aspect of this outbreak was the multidrug resistance profile of the outbreak strain and specifically resistance to kanamycin and gentamicin, and the presence of *lnu(F)*, a determinant of resistance to lincosamides, which are relatively rare for monophasic *S*. Typhimurium in Europe. While not of especial clinical significance as the outbreak strain is susceptible to fluoroquinolones, azithromycin and third-generation cephalosporins which provide effective treatment options for cases of bloodstream infection, the unusual AMR profile constituted an additional characteristic of the outbreak strain to be assessed by all countries in the early stages of investigation for case ascertainment and possible hypothesis generation. This emphasises the usefulness of including more uncommon AMR profiles in early international communications and subsequent incorporation as part of the international outbreak case definitions, where this facilitates identification of possible or probable cases before the application of WGS and/or epidemiological investigations to confirm outbreak cases.

## Conclusion

The large multi-country aspect of this outbreak with multiple products (some marketed under different names) implicated in different countries in children of young age made this outbreak not only unique but one that required an especially urgent, coordinated response. Moreover, the occurrence of contamination in chocolate products in the run-up to Easter when chocolate consumption will increase considerably, especially among children, increased urgency even more.

Early notification of the detection of the outbreak and preliminary findings of the UK investigation followed by rapid multi-country collaboration in information sharing, coordinated and supported by ECDC, was essential to the rapid progress of the outbreak investigations. The descriptive epidemiological information provided strong evidence implicating the vehicle of infection in this outbreak, especially when amalgamated at an international level, sufficient to enable public health and food safety authorities to undertake the rapid, necessary controls. The subsequent information about the detection of the outbreak strain in the implicated processing facility, provided further microbiological confirmation of the link between the company’s products and the Europe-wide outbreak. This demonstrated the utility of and highlighted the need for rapid sharing of microbiological sequence information, derived not just from human disease cases as occurred during this outbreak investigation, but also from, food, animal and environmental sampling.

The control measures taken across all affected countries probably constitute one of the largest chocolate product withdrawals and recalls in European commercial history.

## Supplementary Data

114000 Supplement
