# Supplementary material for: Investigation of an international outbreak of multidrug-resistant monophasic Salmonella Typhimurium associated with chocolate products, EU/EEA and United Kingdom, February to April 2022
Source: Euro Surveill. 2022 Apr 14;27(15):2200314. doi: 10.2807/1560-7917.ES.2022.27.15.2200314 (PMC9012091; doi:10.2807/1560-7917.ES.2022.27.15.2200314)
Supplement: Supplement [file 22-00314_LARKIN_Supplement.pdf]

Supplement

"This supplementary material is hosted by *Eurosurveillance* as supporting information alongside the article *Investigation of an international outbreak of multidrug-resistant monophasic Salmonella Typhimurium associated with chocolate products, EU/EEA and United Kingdom, February to April 2022*, on behalf of the authors, who remain responsible for the accuracy and appropriateness of the content. The same standards for ethics, copyright, attributions and permissions as for the article apply. Supplements are not edited by *Eurosurveillance* and the journal is not responsible for the maintenance of any links or email addresses provided therein."

Further country specific information on the microbiological and environmental investigations of food and control measures undertaken is available in the ECDC and EFSA Rapid Outbreak Assessment on a multi-country outbreak of monophasic Salmonella Typhimurium linked to chocolate products

https://www.ecdc.europa.eu/en/news-events/rapid-outbreak-assessment-multi-country-salmonella-outbreak-linked-chocolate-products

Country specific recall information (not exhaustive):

Belgium:

https://www.favv-afsca.be/consommateurs/rappelsdeproduits/2022/2022-04-05.asp

France:

https://www.economie.gouv.fr/files/files/directions_services/dgccrf/presse/communique/2022/cp-dgccrf-rappel-Ferrero.pdf?v=1649348784

https://www.economie.gouv.fr/files/files/directions_services/dgccrf/presse/communique/2022/CPCompl_information-rappel_Ferrero_07042022.pdf?v=1649682357

https://www.economie.gouv.fr/files/files/directions_services/dgccrf/presse/communique/2022/cp-retrait-rappel-Ferrero080422.pdf?v=1649682357

Germany:

https://www.lebensmittelwarnung.de/bvl-lmw-de/liste/alle/deutschlandweit/10/0

https://www.produktwarnung.eu/wp-content/uploads/2022/04/kiferneu.pdf

Ireland:

https://www.fsai.ie/news_centre/food_alerts/kinder_surprise_recall.html

https://www.fsai.ie/news_centre/food_alerts/kinder_surprise_update.html

Luxembourg:

https://securite-alimentaire.public.lu/fr/actualites/alertes/2022/04/Salmonellose-produits-au-chocolat-Kinder-Ferrero.html

Netherlands:

https://www.nvwa.nl/documenten/waarschuwingen/2022/04/08/update-veiligheidswaarschuwing-enkele-producten-van-ferrero

Norway:

https://www.matportalen.no/verktoy/tilbaketrekkinger/ferrero_kinder_surprise_og_andre_kinderprodukter_trekkes_fra_markedet

Spain:

http://www.aesan.gob.es/AECOSAN/web/seguridad_alimentaria/ampliacion/Kinder.htm

Sweden:

https://www.livsmedelsverket.se/om-oss/press/nyheter/pressmeddelanden/kinderprodukter-aterkallas-av-ferrero-efter-misstanke-om-salmonella

UK:

https://www.food.gov.uk/news-alerts/alert/fsa-prin-22-2022

https://www.food.gov.uk/news-alerts/alert/fsa-prin-22-2022-update-1

https://www.food.gov.uk/news-alerts/alert/fsa-prin-25-2022-update-2
